# Nutritional Status and the Influence of the Vegan Diet on the Gut Microbiota and Human Health

**DOI:** 10.3390/medicina56020088

**Published:** 2020-02-22

**Authors:** Hercules Sakkas, Petros Bozidis, Christos Touzios, Damianos Kolios, Georgia Athanasiou, Eirini Athanasopoulou, Ioanna Gerou, Constantina Gartzonika

**Affiliations:** 1 Microbiology Department, Faculty of Medicine, School of Health Sciences, University of Ioannina, 45110 Ioannina, Greece; pbozidis@uoi.gr (P.B.); kgartzon@uoi.gr (C.G.); 2 Faculty of Medicine, School of Health Sciences, University of Ioannina, 45110 Ioannina, Greece; md05958@uoi.gr (C.T.); md05895@uoi.gr (D.K.); md05845@uoi.gr (G.A.); md05846@uoi.gr (E.A.); md05865@uoi.gr (I.G.)

**Keywords:** vegan, plant-based diet, nutrients, gut microbiota, human health

## Abstract

The human gut microbiota is considered a well-known complex ecosystem composed of distinct microbial populations, playing a significant role in most aspects of human health and wellness. Several factors such as infant transitions, dietary habits, age, consumption of probiotics and prebiotics, use of antibiotics, intestinal comorbidities, and even metabolic diseases may continously alter microbiota diversity and function. The study of vegan diet–microbiota interactions is a rapidly evolving field, since plenty of research has been focused on the potential effects of plant-based dietary patterns on the human gut microbiota. It has been reported that well-planned vegan diets and their associated components affect both the bacterial composition and metabolic pathways of gut microbiota. Certain benefits associated with medical disorders but also limitations (including nutritional deficiencies) have been documented. Although the vegan diet may be inadequate in calorific value, it is rich in dietary fiber, polyphenols, and antioxidant vitamins. The aim of the present study was to provide an update of the existing knowledge on nutritional status of vegan diets and the influence of their food components on the human gut microbiota and health.

## 1. Introduction

During the last decades, plant-based and vegetarian eating patterns proven to be associated with several beneficial health outcomes have been adopted by an increasing proportion of individuals in Western societies [1,2]. Vegetarianism is characterized by a diversity and heterogeneity of dietary practices [3], with the exclusion of certain food groups such as meat, poultry, and similar products, and a focus mainly on fruits, vegetables, grains, pulses, nuts, seeds, and honey. The diet may potentially include seafood (pescetarianism), or eggs (ovo-vegetarianism), dairy products (lacto-vegetarianism), or both (ovo-lacto-vegetarianism) [4,5]. In contrast, the most strictly regimented form of vegetarianism (veganism) is characterized by a complete abstinence of consumption of meat and food of animal origin, such as dairy, eggs, and honey [4], with a diet consisting solely of plant foods like grains, vegetables, fruits, legumes, nuts, seeds, and vegetables fats and oils [6].

Veganism is usually adopted as the result of ethical principles related to animal rights and welfare, since the way these products are acquired is considered violent and barbaric [4,7], but also due to spiritual, moral, and religious values [3,8], socioeconomic considerations [9], and environmental concerns as well, focusing on the energy and natural resources savings in food production [5,10]. Thus, disparities in the prevalence rates of both vegetarianism and veganism have been observed in data reported across several countries, but also between different territories within the same country. Although the percentage of vegans has increased by 350% during the last decade [4], today only 0.1% to 1% of the adult population in Germany claims to follow a vegan diet [11,12]. In contrast, the constantly increasing prevalence of vegetarianism and veganism within North Americans has been reported to be as high as 5% and 2%, respectively [8], whereas the prevalence of veganism among US individuals with specific religious beliefs was reported to be 7.6% [13]. Other studies involving countries worldwide have yielded variable prevalence rates of vegetarianism within the general population: 0.77% in China [14], 0.79% in Italy [15], 1.5% in Spain [16], 3.3% in Germany [11], 3.8% in Norway [17], 4.1% in Finland [18], from 3% to 5% in Latvia [19], up to 11.2% in Australia [20], 33% in South Asia [21], and from 4.8% to 15.6% in Sweden [17]. In addition, vegetarian diets have also become popular due to potential health benefits among adolescents and young adults, especially females; in a recent report, prevalence rates from 8% to 37% and from 1% to 12% in female and male Australian teenagers were quoted, respectively [22].

Much research has been focused on the potential effects of veganism on health and wellness. Certain benefits associated with multiple medical comorbidities but also limitations such as nutritional deficiencies with respect to vitamins, minerals, and proteins have been reported [1,3,4,18,20,23,24]. Veganism has been widely accepted as the prototype of healthy diet related to gut microbiota [25,26,27], cardiovascular disease [24], diabetes, cancer, chronic kidney disease, cataracts, obesity, normal pregnancy outcomes [1,3,8,9,28,29,30], metabolic syndrome, the brain [2], bone health [31], and more. It has been reported that vegans demonstrated a risk reduction of 75% for hypertension, 47%–78% for type 2 diabetes mellitus, and 14% for total cancer incidence [8]. In addition, several studies have demonstrated that plant-based diets have been associated with reductions in mortality rates [13], although much research is needed on the long-term health of consumers [32]. In addition, many people interested in vegan diets also adopt generally healthy lifestyle habits including regular physical activity, abstinence from smoking and alcohol [3,33], consequential social interactions, emotional regulation, and cognitive and behavioral investments [34]. Nowadays, successful social media campaigns focus on the visibility and acceptance of veganism among athletes and people working in health and fitness-related fields [35]. The aim of the present study was to provide an update of the existing knowledge on nutritional status in vegan diets and their influence on human gut microbiota and health.

## 2. Vegan Nutrition

The vegan diet does not include products made out of animals; thus, most of the nutrient income is based on the lower levels of the food pyramid. This kind of nutrition includes high intake of fruits and vegetables and low intake of both sodium and saturated fat [20]. Apart from the nutrients, plants contain numerous phytochemicals, including carotenoids and polyphenols. Such compounds are polyphenols found in grapes, berries, and nuts, indole-3-carbinol in cruciferous vegetables such as sprouts, cabbage, and cauliflower, isoflavones found in legumes, including clover, soy, and lupine, and lycopene in tomatoes. In general, these substances, which are referred as food ingredients, have no additive nutritional value, but they can affect various metabolic pathways of the body, providing multiple health benefits [36,37,38,39]. However, if a vegan diet is not appropriately planned, reduction of caloric intake and nutritional deficiency of fatty acids, proteins, vitamins, and minerals may appear [1].

### 2.1. Macronutrients

Carbohydrates may be subdivided into digestible and indigestible compounds. Plant-based diets composed of fiber-rich foods refer to indigestible carbohydrates also called “dietary fiber”, including non-starch polysaccharides, lignin, resistant starch, and non-digestible oligosaccharides [40]. These macronutrients, that are intrinsic and intact in plants [41], are also resistant to digestion in the small intestine and pass into the large intestine, where they are fermented and produce specific bacterial metabolites, such as short-chain fatty acids (SCFAs), associated with beneficial effects [40]. Plant foods that are rich in fiber include whole grains, vegetables, fruits, and legumes. Dietary fiber appears to confer benefits to various aspects of human health: cardiovascular disease, body weight management, immunity, and intestinal health including colorectal cancer prevention, laxation, regularity, and appetite control (satiation, satiety) [41]. In particular, prebiotics such as oligosaccharides of natural (e.g., human milk oligosaccharides) or synthetic origin (e.g., galacto-oligosaccharides, fructo-oligosaccharides), phytochemicals, polyphenols and derivatives, carotenoids, and thiosulphates exert several beneficial effects [42]. These effects include increases in bifidobacteria, lactobacilli, and calcium absorption, decreases in other bacteria populations and protein fermentation, improvement in gut immunity, production of beneficial metabolites, and effects on gut barrier permeability [43].

The content in fatty acids and saturated fats is particularly low in a plant diet, leading to weight loss, improved lipid profile, and reduced blood pressure, associated with prevention of coronary heart disease and other chronic diseases [44,45,46]. Plant foods contain just small amounts of monounsaturated and polyunsaturated fatty acids, mainly α-linolenic acid (ALA), and therefore omega-3 polyunsaturated fatty acids can be obtained from most vegetable oils, cereals, walnuts, chiaseed, rapeseed, linseed, camelina, canola, and hemp [4,45,47,48]. Microalgae supplements containing docosahexaenoic acid (DHA), as well as DHA-fortified foods, regular supplies of ALA foods, and supplements are also good sources of essential fatty acids [1].

One of the major concerns about the vegan diet is the lack of protein intake providing the lowest energy for body functions when comparing to vegetarians and meat consumers [49]. The quality of a protein is determined by the digestive efficiency and the content of essential amino acids. High digestibility is provided by purified or concentrated vegetable proteins such as soy and gluten, while the majority of the vegetable products are characterized by low digestibility. It has been well documented that the presence of plant cell wall and antinutritional agents (enzyme inhibitors, tannins, phytates, glucosinolates, isothiocyanates), as well as food processing and heat treatment, may be inhibitory factors in protein digestibility [4,50]. In general, if certain plant foods are consumed in appropriate combinations, they can provide all the essential amino acids for human nutrition, although some of them may be absent in certain plants, including lysine in cereals, rice, and corn, and methionine in legumes [4]. Vegans usually include sufficient amounts of legumes in their diet, a protein source that has been reported as a potential preventive factor against stomach, prostate, and colon cancer [1]. In addition, consumption of legumes may demonstrate a cardioprotective effect by decreasing the levels of circulating serum lipids and lipoproteins including total cholesterol, low-density lipoprotein (LDL), and triglycerides [51].

### 2.2. Micronutrients

Although the vegan diet may have inadequate calorific value, it is rich in antioxidant vitamins and phytochemicals. A minimal amount of vitamins is usually required for metabolic and homeostasis functions [20]. Plant foods clearly supply vitamins to this kind of diet, including vitamin C (L-ascorbic acid) and carotenoids. Carotenoids are precursors of vitamin A, such as β-carotene or provitamin A, which is found in abundance in carrots. Polyunsaturated vegetable oils contain significant amounts of liposoluble vitamin E. Selenium, a trace element which is very important for the production of glutathione peroxidase, is also found in many plant foods [52,53]. Vitamins also appear to have a protective role in various neoplastic diseases such as hematological (vitamin C), glioma, lung (vitamin A), prostate, breast, colorectal (vitamin E and selenium), oropharyngeal, bladder, skin, uterine, and ovarian cancers (selenium) [53].

In contrast, there are significant deficiencies concerning other vitamins, including vitamin B12 and vitamin D. Vitamin B12 is a water-soluble vitamin that is found predominantly in products of animal origin, playing a vital role in hematopoiesis and nervous system, whereas a severe deficiency may occur by either alterations in absorption or nutritional insufficiency [23,54,55], resulting in several comorbidities such as megaloblastic anemia, stroke, Alzheimer’s and Parkinson’s diseases, vascular dementia, cognitive impairment, and more [2]. In order to prevent vitamin deficiency due to inadequate dietary intake, there is an urgent need for vegans to incorporate reliable vitamin B12 sources including vitamin B12-fortified foods such as fortified soy and rice beverages, certain breakfast cereals, or vitamin B12 dietary supplements which usually provide high absorption capacities [1,4,18,24,31,50]. Other sources of vitamin B12 include vegetables like broccoli, asparagus, and bean sprouts, specific types of nutritional mushrooms, tea leaves, tempeh, edible algae including dried green laver (*Enteromorpha* spp.) and purple laver (*Porphyra* spp.), other microalgae (klamath, *Chlorella*), and cyanobacteria (spirulina, *Nostoc*). However, the vitamin content may vary among these products since many of them contain only traces of vitamin B12 and should not be considered as an adequate source for the daily intake [6,15,20]. High prevalence rates of vitamin B12 deficiency (up to 80%) have been reported among Hong Kong and Indian populations, where vegans rarely include fortified foods or supplements in their diets [24].

Vitamin D, related to both calcium absorption and bone mineralization, plays an essential role in bone health [31]. Its levels depend predominantly on adequate sun exposure, and thus supplementation might not be necessary, especially among individuals living in low latitude regions. Low 25-hydroxyvitamin D concentrations in the serum have been documented in vegan societies, especially in winter or spring, or in those living in high latitudes [6,43,56]. Vitamin D3 (cholecalciferol) can originate from plants or animals, whereas vitamin D2 (ergocalciferol) is produced by the action of ultraviolet radiation. Mushrooms treated under ultraviolet light can be an important source of vitamin D [31,45]. Alternative vitamin D sources are breakfast cereals and nondairy substitutes for milk other than soy, like oat, almond, and rice drinks [6]. If sun exposure and intake of fortified foods are insufficient to meet the nutrients requirements, vitamin D supplements are recommended, both for children and adults [18,57].

Deficiencies in minerals such as iodine, calcium, and zinc may also occur. Iodine deficiency is very common among vegans, often leading to acquired hypothyroidism [58]. Vegan sources of iodine include iodized salt and sea vegetables containing various amounts of the mineral [45]. There are abundant plant-based sources of calcium; however calcium bioavailability is inversely proportional to the amounts of oxalate, and to a lesser extent, to phytate and fiber found in vegetables [45,50]. High-calcium foods include several green leafy vegetables, tofu, tahini [1], and fortified foods such as cereals, soy, rice, almond and coconut beverages, orange and apple juices, and to a lesser extent unsweetened cranberry and low sodium tomatoes [59]. Nevertheless, the best absorption is provided by low-oxalate vegetables, including broccoli, kale, turnip greens, Chinese cabbage, and bok choy [60].

Vegans have the opportunity to consume as much iron as non-vegans daily. However, both iron and ferritin levels in the blood are lower in vegans than in non-vegans. The absorption of iron derived from heme is significantly higher compared to non-heme iron intake from plant foods. This can be counteracted by consuming ascorbic acid (citrus, strawberries, kiwi), a component necessary for the absorption of non-heme iron [1,50]. Legumes, beans, whole grains, integral cereals, dark-green leafy vegetables, fruits, seeds, and nuts can be used as sources of iron [16,30,61]. Zinc acts as a catalyst in iron metabolism and is not as easily absorbed from plant sources as it is from animal products, which usually supply half of the zinc intake [4]. In vegans, low plasma zinc levels can lead to iron deficiency anemia. Zinc-rich plant foods are wholemeal bread, peas, corn, nuts, carrots, whole grains, wheat germs, soybeans, cabbage, radish, watercress, and legumes [4,30,62].

## 3. Influence of Vegan Diets on the Human Gut Microbiota

### 3.1. Gut Microbiota Composition and Functional Aspects

The microbial composition of the human gut microbiota consists of several taxa of microorganisms, such as bacteria, viruses, protozoa, and fungi [63]. It is estimated that the human gastrointestinal tract harbours approximately 100 trillion microorganisms, comprising more than 1000 bacterial species [63,64]. Bacteroidetes, Firmicutes, Actinobacteria, Proteobacteria, Fusobacteria, and Verrucomicrobia are primarily found as part of the normal gut flora, where Bacteroidetes and Firmicutes represent the 90% of total bacterial phyla constitution, and Actinobacteria, Proteobacteria, and Verrucomicrobia are represented to a lesser extent [64,65,66]. A brief summary of commonly encountered bacteria in the gut microbiota is given in Table 1.

Several factors such as infant transitions (birth gestational age, type of delivery, milk-feeding practices, infant weaning), dietary habits, age, ethinicity, cultural and lifestyle habits (exercise, alcohol consumption), geographic and environmental factors, stress, obesity, consumption of probiotics and prebiotics, use of antibiotics, intestinal comorbidities, and metabolic diseases, may continuously alter bacterial composition and diversity [26,27,63,67,68]. Studies in vivo have reported that changes in gut microbiota composition have been shown to exert an important role in maintaining the function of the intestinal barrier. Indeed, low-fiber, high-protein, and high-fat diets have been documented to increase both intestinal inflammation and permeability by altering the translocation of bacterial populations and metabolites that modulate inflammation [69]. In addition, metabolites derived from gut microbiota including bacteriocins, SCFAs, microbial amino acids, and vitamins seem to play a vital role in activating the intestinal immune response thus defending against external pathogens [70]. Recently, the term “metabolic endotoxemia” was introduced to describe a significant increase in bacterial lipopolysaccharide (LPS) plasma levels observed both in animals and humans in high-fat diets [71,72]. Under such conditions, the increase in LPS plasma levels, caused by an imbalance in the homeostasis of the microbiota, induces a low intensity systemic inflammation which has shown to be associated with obesity, diabetes, and insulin resistance [73]. Although there is a variety of functional competencies among different intestinal microbial communities, the normal gut microbiota, which is considered the largest organ and the most complex system of microorganisms [74], plays a crucial role in most of the human health aspects and well-being, including digestion of foods, metabolic breakdown of drugs and toxins [75], nutrient metabolism [76,77], antimicrobial protection [26], development and homeostasis of immunity [75,78], the gut–brain axis [79,80], the gut–liver axis [74,81], and gastrointestinal and cardiovascular health [26,82,83]. Today, high-throughput microbiome sequencing technologyies including 16S rRna gene sequencing, whole genome metagenomics, metatranscriptomics, metaproteomics, and metabolomics, offer the most considerable insight into the gut microbiota ecosystem and their metabolic functions [68].

**Figure 1:**
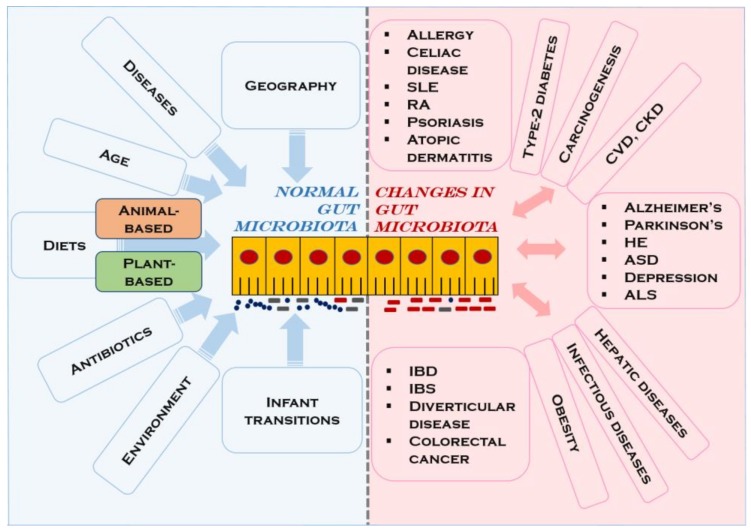
Alterations in the composition of the gut microbiota and associated clinical disorders. SLE, systemic lupus erythematosus; RA, rheumatoid arthritis; CVD, cardiovascular disease; CKD, chronic kidney disease; HE, hepatic encephalopathy; ASD, autism spectrum disorder; ALS, amyotrophic lateral sclerosis; IBD, inflammatory bowel disease; IBS, irritable bowel syndrome.

An imbalance or alteration in microbial composition and activity, also called “gut microbiota dysbiosis”, has been associated with several clinical manifestations, although it is not yet clear if dysbiotic patterns are the cause or the consequence of the disease [84]. These disorders include obesity, type-2 diabetes mellitus, neurological and neuropsychiatric cormobidities (Alzheimer’s and Parkinson’s diseases, hepatic encephalopathy, autism spectrum disorder, depression, amyotrophic lateral sclerosis), allergy, carcinogenesis, autoimmune diseases (celiac disease, systemic lupus erythematosus, rheumatoid arthritis, psoriasis, atopic dermatitis), infectious diseases (*Clostridium difficile* infection), cardiovascular disease, and chronic kidney, hepatic, and gastrointestinal diseases [63,66,74,75,81,85,86,87,88]. Among the most common disorders of the gastrointestinal tract related to gut microbiota dysbiosis are the two major types of inflammatory bowel disease, ulcerative colitis and Crohn’s disease [75]. Irritable bowel syndrome, diverticular disease, and colorectal cancer have also been reported [74] (Figure 1).

### 3.2. Impact of Vegan Food Components on the Human Gut Microbiota

It has been well documented that long-term dietary patterns can alter both diversity and function of the gut microbiota, while it is not well known how the short-term consumption of different diets may alter changes in the gut microbiota composition and functionality [82,89]. Food polymers, including fibers, polyphenols, fats, and proteins are commonly involved in main gut microbiota metabolic pathways [79]. Omnivore, ovo-lacto vegetarian, and vegan diets are sources of nutrients for microorganisms and they have also their own microbiota, conferring heterogeneous effects on both abundance and diversity of the gut microbiota [67]. Vegan and vegetarian gut microbiota profiles may not differ and both include a greater profusion of beneficial bacteria when compared to that of omnivores. On the contrary, the human gut microbiota appears to be altered with a greater impact in omnivores than in vegans, and is composed of bile-tolerant potentially harmful microorganisms, since animal-based diets are usually characterized by increased levels of fecal bile acids [25]. Bile acids, which are cholesterol-derived compounds synthesized in hepatocytes, enable the emulsification of dietary fats and the intestinal absorption of lipids and lipofilic vitamins, act in several metabolic and inflammatory pathways, and alter the composition of gut microbiota through farnesoid X receptor and G protein-coupled membrane receptor 5 directly and indirectly [90,91]. Moreover, since the occurrence and abundance of antimicrobial resistance genes have been found significantly lower in gut microbial communities of vegans than those of omnivores, animal-based diets may be involved in antimicrobial resistance spread within the gut microbiota environment [92].

Characterization of the human gut bacterial diversity is usually determined by using enterotyping, interpreted as a bacterial *Prevotella* to *Bacteroides* ratio (P/B) [93]. Although the gut microbiota structure in strict vegans has not been precisely specified, and several environmental, cultural and genetic factors have been associated with Western to non-Western gut community differentiation, it has been reported that the ratio P/B was higher in persons with a natural fiber and starch intake than in individuals following a Western-type diet [94,95]. Thus, gut microbiota are dominated by *Prevotella* species in persons with plant-based dietary habits, such as populations living in African, Asian, and South American societies, while *Bacteroides*-driven enterotype is predominant in individuals living in Western societies that consume diets rich in animal protein, amino acids, and saturated fats [75,93]. Interestingly, *Prevotella* spp. have been found to provide effective anti-inflammatory properties on certain diseases [26] including inflammatory arthritis [96] and multiple sclerosis [97], whereas *Bacteroides* spp. are usually involved in several infections providing antimicrobial resistance to a variety of antibiotics, and may act as useful commensals to the human host as well [98].

**Figure 2:**
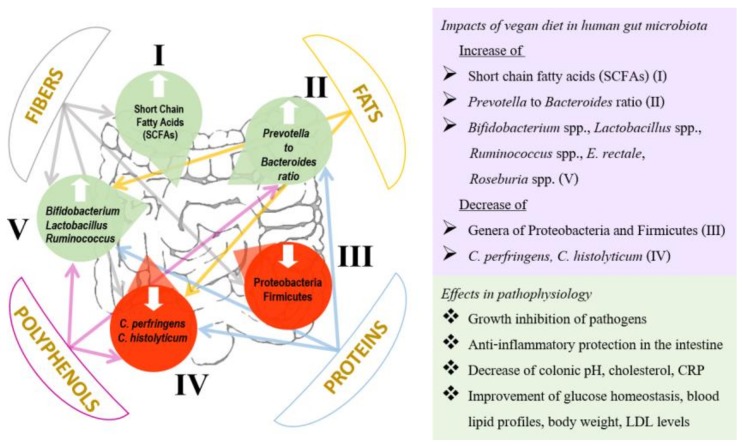
Impact of vegan food components in the human gut microbiota. *E. rectale: Eubacterium rectale; C. perfringens: Clostridium perfringens; C. histolyticum: Clostridium histolyticum.* LDL: low-density lipoprotein; CRP: C-reactive protein.

Dietary fiber may influence the gut microbial community in terms of type, number, and consistency of bacterial species. Thus, indigestible carbohydrate diets rich in whole grain and wheat bran are associated with an increase of *Bifidobacterium* spp. and *Lactobacillus* spp., whereas resistant starch and whole grain barley may also increase lactic acid bacteria including *Ruminococcus* spp., *Eubacterium rectale*, and *Roseburia* spp. It is difficult to state the same for other members of the Firmicutes phylum such as *Clostridium* and *Enterococcus* species, which are both reduced [26,87]. Both bifidobacteria and lactobacilli demonstrate an exclusive potential of saccharolytic metabolism and have been considered to be associated with a protective role in the human gut barrier by inhibiting the invasion and growth of bacterial pathogens [26,41] (Figure 2). *Akkermansia muciniphila,* a mucin-degrading bacterium of the intestinal microbiota which may represent 3%–5% of the total microbial community in healthy subjects, has also been related to the enhancement of gut barrier function, prevention of gut bacterial translocation, inflammation, obesity, intestinal homeostasis, and metabolism [99]. Animal studies have shown that prebiotic supplementation may consistently promote the abundance of this bacterium in the gut [100]. Further studies in animals have shown that a purified membrane protein from *A. muciniphila* or the pausterized bacterium improves metabolism in obese and diabetic mice [101]. Recently, an exploratory-study in obese and overweight human volunteers showed the beneficial effect associated with *A. muciniphila* supplementation [102]. In addition, studies in mice showed that when comparing the intake of a crude fraction of wheat bran and the same fraction with reduced particle size and wheat bran-derived arabinoxylan oligosaccharides, only the crude fraction of wheat bran was followed by an increase in *Akkermansia* spp. on gut microbiota, thus providing beneficial effects in the context of obesity [103]. Apart from the different impact on gut microbiota composition, the intake of crude fraction of wheat bran with reduced particle size also led to the observation of hepatic anti-inflammatory effects [104].

Fermentable dietary fiber has been shown to serve as a substrate for intestinal bacteria metabolism. The end products of the bacterial metabolism include certain metabolites such as SCFAs [65]. The main SCFAs include acetate and propionate (used as substrates for lipid, glucose, and cholesterol metabolism), and butyrate (which plays a key role in immunoregulation and maintainance of tissue barrier function), serving as energy substrates for the gut epithelial cells [40,83]. They probably provide anti-inflammatory effects in the intestine [87]. They are also involved in several other important physiological functions, including decrease of colonic pH and circulating cholesterol, improvement of glucose tolerance and insulin sensitivity, growth inhibition of emerging Enterobacteriaceae pathogens (*Salmonella* spp., adherent-invasive *Escherichia coli*), stimulation of water and sodium absorption, energy provision to the colonic epithelial cells, inhibition of cancer cell proliferation by interfering with multiple mechanisms, and prevention of high-fat diet induced obesity by stimulating fat oxidation [66,68,84,105]. Therefore, SCFAs improve blood lipid profiles, glucose homeostasis, and body composition, reduce body weight [93], strengthen the mucosal barrier [87], and act protectively against several disorders including type 2 diabetes mellitus, inflammatory bowel disease, and immune diseases [26] (Figure 2).

Apart from fibers, polyphenols, which are also abundant in vegan diets, increase both *Bifidobacterium* spp. and *Lactobacillus* spp., providing cardiovascular protection as well as antibacterial and anti-inflammatory effects [26]. Most of these compounds exhibit structural diversity, and consist of flavonoids, phenolic acids, stilbenes, lignans, and secoiridoids. They pass into the colon and are metabolized by colonic bacteria which influence their bioactivity, while a tiny proportion is possibly absorbed in the small intestine [77]. Fruits such as grape, blueberry, sweetsop, mango, and citrus, vegetables, medicinal plants, microalgae, herbs, seeds, cereals, and beverages including coffee, tea, cocoa and red wine are good sources of polyphenols [40]. Beneficial interactions between tea or soy isoflavones and intestinal microbiota have been reported, whereas wild blueberries—a good source of polyphenols—have been shown to increase *Bifidobacterium* and *Lactobacillus* species [106]. A decrease in pathogenic *Clostridium perfringens* and *Clostridium histolyticum* is probably attributable to the consumption of fruit, seed, tea, and wine polyphenols [87]. It has also been reported that proanthocyanidin-rich extract from grape seeds increased the number of *Bifidobacterium* spp. significantly, while genera of Enterobacteriaceae family were decreased [107]. In an another study the consumption of red wine was associated with an increase of bifidobacteria and species of the *Enterococcus, Bacteroides,* and *Prevotella* genera, whereas nonbeneficial bacteria such as *Clostridium* spp. were inhibited, providing possible prebiotic benefits of red wine polyphenols and resulting in the reduction of both cholesterol and C-reactive protein (CRP) [108]. A significant increase of high-density lipoproteins and decrease of CRP and triglyceride serum levels have also been reported after consumption of cocoa-derived polyphenols [87] (Figure 2).

Fats are considered to be an efficient source of energy, and on the basis of current data, both the quality and quantity of the dietary fat intake may influence the gut microbiota composition [65]. Vegan diets are low-fat diets containing monounsaturated and polyunsaturated fats, altering the microbial intestinal composition by increasing the Bacteroidetes to Firmicutes ratio. On the contrary, animal saturated fats increase genera of Proteobacteria and Firmicutes and also decrease *Bifidobacterium* spp., which may provoke inflammation, leading gradually to metabolic derangements [26] (Figure 2). There is a strong and consistent evidence that the consumption of animal-fat diets can be a major driving factor in cardiovascular disease pathogenesis through the increase of both total serum cholesterol and LDL levels [87].

The protein-energy status in vegans has been reported lower when compared to omnivores [109]. Studies examining the impact of dietary proteins on the microbiota confirmed that both *Bifidobacterium* and *Lactobacillus* species as well as the intestinal SCFA levels were increased after the consumption of pea protein, while both pathogenic *C. perfringens* and *Bacteroides fragilis* were decreased [87]. The beneficial effect of the consumption of walnuts on the gut microbiota composition by increasing *Ruminococcus* spp. and *Bifidobacterium* spp. and decreasing *Clostridium* spp. has also been reported [26] (Figure 2). In contrast, animal protein intake appears to have a significant role in the pathogenesis of inflammatory bowel disease since it may alter gut microbiota composition by increasing *Bacteroides* spp., *Alistipes* spp., and *Bilophila* spp., and decreasing beneficial *Lactobacillus* spp., *Roseburia* spp., and *E. rectale* [87]. In addition, diets with high animal protein intake are associated with cardiovascular disease, since the consumption of red meat may alter the gut microbiota composition resulting in the production of a proatherogenic metabolite (trimethylamine-N-oxide) in mice [110].

Among micronutrients, certain vitamins including vitamin K and B-complex vitamins (biotin, cobalamin, folate, nicotinic acid, panthotenic acid, pyridoxine, riboflavin, thiamin), all involved in bacteria metabolism, can be synthesized in gut microbiota [77]. On the other hand, studies performed in human volunteers showed that carotenoids such as blackcurrant lutein were found to affect microbiota composition by increasing *Bifidobacterium* spp. and *Lactobacillus* spp., and reducing *Bacteroides* spp. and *Clostridium* spp. [111].

## 4. Conclusions

Vegan diets have been gaining in popularity among Western societies in recent years, as several clinical disorders and malignancies caused by the consumption of animal-based products still occur frequently in developed countries. The evaluation of such comorbidities may reveal further modes of pathogenesis, including the consumption of such diets. The often-claimed effect of plant-based diets on human health is attributed to the activity of major nutritional components on conferring health benefits to the host. Such components include dietary fiber, monounsaturated and polyunsaturated fats, proteins, polyphenols, and micronutrients. Nevertheless, one of the major concerns about the vegan diet is the nutritional status restriction of certain nutrients like proteins and fats. Thus, vegans should always follow a comprehensive diet plan in order to avoid the lack of essential nutrients. It has been well documented that different factors may contribute to the gut microbiota composition and variation. The gut microbiota is composed of highly diverse microbial communities which interact and compete for such nutrients, producing metabolites associated with most aspects of human health and well-being. Vegan diets and their main components affect both bacterial composition and metabolic pathways of gut microbiota by increasing beneficial microorganisms. However, more studies are needed to determine the impact of these diets on gut microbiota. Further, a better understanding of the individualized nature and diversity of gut microbiota may help explain disease susceptibility and will lead to new approaches in the medical field.

## Figures and Tables

**Table 1:** Normal human gut microbiota composition.

Bacteroidetes	Firmicutes	Actinobacteria
*Bacteroides*	*Clostridium*	*Bifidobacterium*
*Prevotella*	*Faecalibacterium*	
	*Enterococcus*	**Verrucomicrobia**
**Proteobacteria**	*Streptococcus*	*Akkermansia*
*Escherichia*	*Roseburia*	
*Shigella*	*Lactobacillus*	**Fusobacteria**
	*Bacillus*	*Fusobacterium*
	*Eubacterium*	
	*Ruminococcus*	
